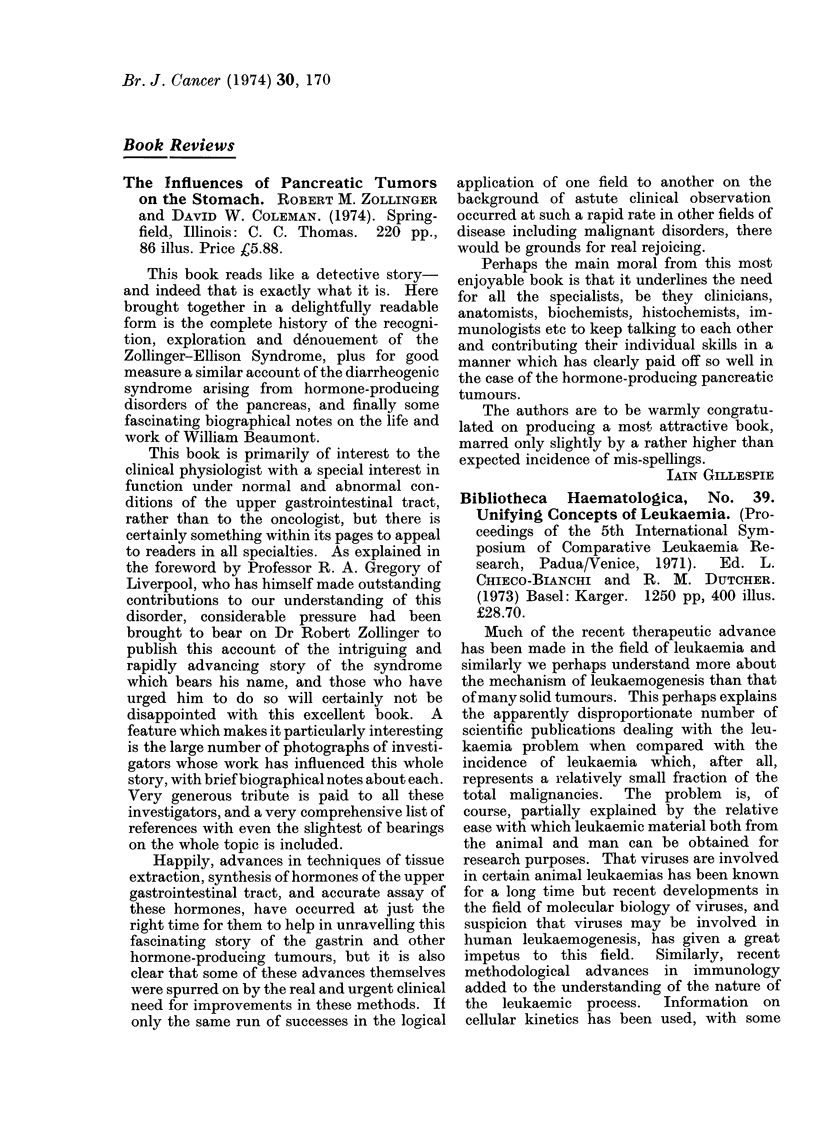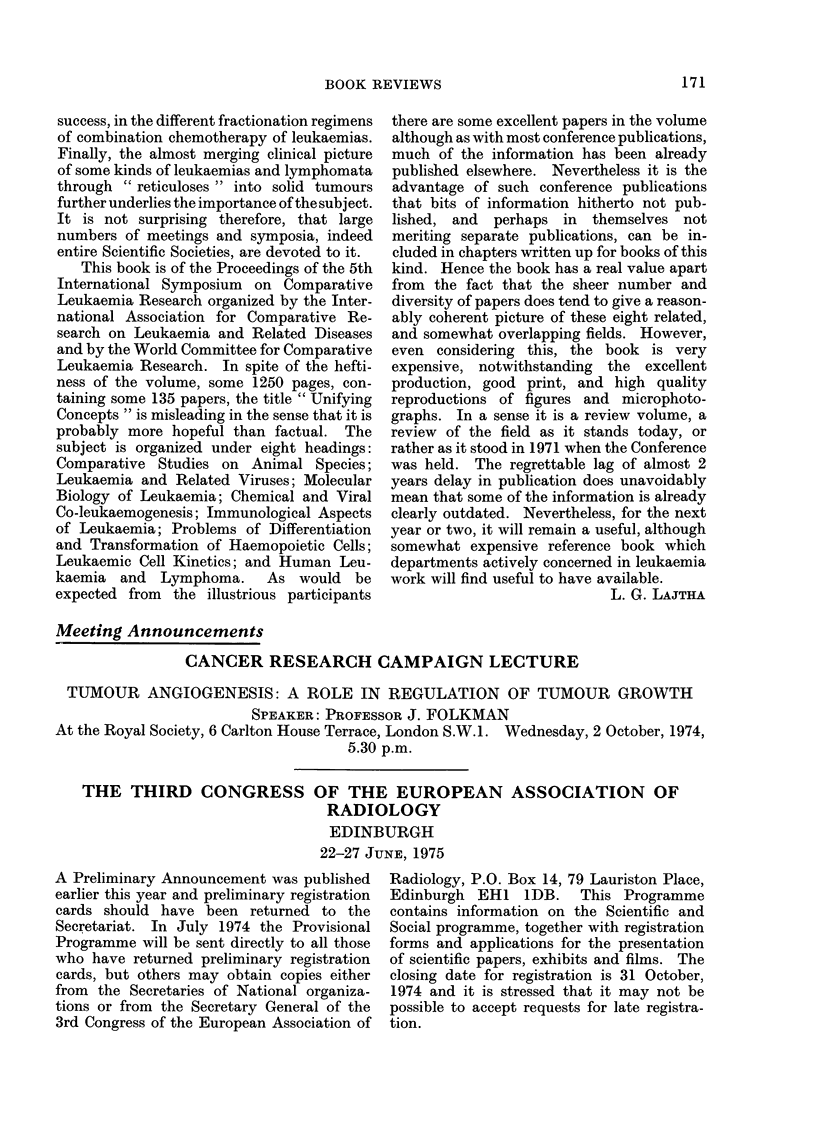# Bibliotheca Haematologica, No. 39. Unifying Concepts of Leukaemia

**Published:** 1974-08

**Authors:** L. G. Lajtha


**Bibliotheca Haematologica, No. 39. Unifying Concepts of Leukaemia.** (Proceedings of the 5th International Symposium of Comparative Leukaemia Research, Padua/Venice, 1971). Ed. L. CHIECO-BIANCHI and R. M. DUTCHER. (1973) Basel: Karger. 1250 pp, 400 illus. £28.70.

Much of the recent therapeutic advance has been made in the field of leukaemia and similarly we perhaps understand more about the mechanism of leukaemogenesis than that of many solid tumours. This perhaps explains the apparently disproportionate number of scientific publications dealing with the leukaemia problem when compared with the incidence of leukaemia which, after all, represents a relatively small fraction of the total malignancies. The problem is, of course, partially explained by the relative ease with which leukaemic material both from the animal and man can be obtained for research purposes. That viruses are involved in certain animal leukaemias has been known for a long time but recent developments in the field of molecular biology of viruses, and suspicion that viruses may be involved in human leukaemogenesis, has given a great impetus to this field. Similarly, recent methodological advances in immunology added to the understanding of the nature of the leukaemic process. Information on cellular kinetics has been used, with some success, in the different fractionation regimens of combination chemotherapy of leukaemias. Finally, the almost merging clinical picture of some kinds of leukaemias and lymphomata through " reticuloses " into solid tumours further underlies the importance of the subject. It is not surprising therefore, that large numbers of meetings and symposia, indeed entire Scientific Societies, are devoted to it.

This book is of the Proceedings of the 5th International Symposium on Comparative Leukaemia Research organized by the International Association for Comparative Research on Leukaemia and Related Diseases and by the World Committee for Comparative Leukaemia Research. In spite of the heftiness of the volume, some 1250 pages, containing some 135 papers, the title " Unifying Concepts " is misleading in the sense that it is probably more hopeful than factual. The subject is organized under eight headings: Comparative Studies on Animal Species; Leukaemia and Related Viruses; Molecular Biology of Leukaemia; Chemical and Viral Co-leukaemogenesis; Immunological Aspects of Leukaemia; Problems of Differentiation and Transformation of Haemopoietic Cells; Leukaemic Cell Kinetics; and Human Leukaemia and Lymphoma. As would be expected from the illustrious participants there are some excellent papers in the volume although as with most conference publications, much of the information has been already published elsewhere. Nevertheless it is the advantage of such conference publications that bits of information hitherto not published, and perhaps in themselves not meriting separate publications, can be included in chapters written up for books of this kind. Hence the book has a real value apart from the fact that the sheer number and diversity of papers does tend to give a reasonably coherent picture of these eight related, and somewhat overlapping fields. However, even considering this, the book is very expensive, notwithstanding the excellent production, good print, and high quality reproductions of figures and microphotographs. In a sense it is a review volume, a review of the field as it stands today, or rather as it stood in 1971 when the Conference was held. The regrettable lag of almost 2 years delay in publication does unavoidably mean that some of the information is already clearly outdated. Nevertheless, for the next year or two, it will remain a useful, although somewhat expensive reference book which departments actively concerned in leukaemia work will find useful to have available.

L. G. LAJTHA